# PbsB Regulates Morphogenesis, Aflatoxin B1 Biosynthesis, and Pathogenicity of *Aspergillus flavus*

**DOI:** 10.3389/fcimb.2018.00162

**Published:** 2018-05-17

**Authors:** Jun Yuan, Zhong Chen, Zhiqiang Guo, Ding Li, Feng Zhang, Jiaojiao Shen, Yi Zhang, Shihua Wang, Zhenhong Zhuang

**Affiliations:** Key Laboratory of Pathogenic Fungi and Mycotoxins of Fujian Province, Key Laboratory of Biopesticide and Chemical Biology of Education Ministry, School of Life Sciences, Fujian Agriculture and Forestry University, Fuzhou, China

**Keywords:** pbsB, aflatoxin B1, MAPKK, crop invasion, *Aspergillus flavus*

## Abstract

As an opportunistic pathogen, *Aspergillus flavus* is one of the major causes of food contamination around the world. In this study, *pbsB* gene knockout mutant (Δ*pbsB*) and *pbsB* overexpression strain (OE) of *A. flavus* were constructed by homologous recombination. The results showed that the mycelia growth, conidiation, and the formation of sclerotia in Δ*pbsB* mutant were significantly suppressed, and up-regulated in OE strian compared to wild-type strain (WT). Q-PCR analysis showed that PbsB regulated the sclerotia formation through sclerotia related gene *nsdC*. With TLC and qRT-PCR analysis, it was found that PbsB up-regulated the bio-synthesis of aflatoxin B1 (AFB1) through regulatory gene *aflR* and structural gene *aflC, aflD, aflK*, and *aflQ* in the aflatoxin gene cluster. In osmotic stress response analysis, Δ*pbsB* mutant was significantly more sensitive to osmotic pressure with 1.2 mol/L sorbitol, compared to WT and OE strains. In virulence analysis, the infection capacity of Δ*pbsB* strain to peanut and maize kernels decreased dramatically, and significantly fewer spores and lesser mycelia were produced in Δ*pbsB* strain on the surface of peanut and maize kernels, and the infection capacity of OE strain to kernels increased significantly compared with WT strain. The AFB1 bio-synthesis ability of *A. flavus* in crop invasion models was also found to be coincide with the expression level of *pbsB*. All the results of the study shows that, as a MAPKK, PbsB is critical for growth and virulence in *A. flavus*, and lay a theoretical foundation for the prevention and control of *A. flavus* contamination.

## Introduction

The soil-borne pathogen *Aspergillus flavus* is an opportunistic pathogen, which is one of the major causes of the mycotoxins contamination to crops (such as peanuts, maize kernel, and cotton) around the world (Satterlee et al., 2016). The most agriculturally important mycotoxins known are aflatoxins, in which aflatoxin B1 (AFB1) is the most toxic, carcinogenic, mutagenic, and teratogenic secondary metabolite (Xing et al., 2016). AFB1 has been classified by the International Agency for Research on Cancer (IARC) as a Group 1 carcinogen (Wu et al., 2014). AFB1 is extensively linked to liver cancer, reports indicated that chronic exposure to AFB1 through daily diets lead to immunosuppression, fatty liver, hepatic lesions, and even hepatomas (Yu, 2012). And high dose aflatoxins could cause death from aflatoxicosis (Misihairabgwi et al., 2017).

The development and secondary metabolite production of filamentous fungi are found to be regulated by a number of orthodox regulatory factors (Cary et al., 2012). For example, the global regulator, VeA, mediates aflatoxin production via AflR, and regulates the development of cleistothecia in *Aspergillus nidulans* and sclerotia in *A. flavus* (Cary et al., 2006). Environmental factors (such as nutrition and stresses) are also found to be involved in the morphogenesis and secondary metabolites bio-synthesis in *Aspergillus* spp. (Han et al., 2003; Fountain et al., 2016). MAP (mitogen-activated protein) kinase cascade is one of the mechanisms for eukaryotic cell to transfer the extracellular environment information through plasma membrane-associated receptors to the expression of target genes in the nucleus. Exposure of budding yeast to increased extracellular osmolarity activates one of the MAP kinase cascades—high osmolarity glycerol (HOG) response pathway, in which the MAP kinase (MAPK) HOG1 is activated with the phosphorylation of Thr174 and Tyr176 by MAP kinase kinase (MAPKK) Pbs2 (Ferrigno et al., 1998). The activation of Pbs2 requires to be phosphorylated by a MAP kinase kinase kinase (MAPKKK) STE11, or one of two partially redundant MAPKKKs, SSK2, and SSK22 (Posas et al., 1998). *A. nidulans pbsB* or *hogA* (*pbs2p* or *hog1* in yeast) deletion mutant showed growth inhibition under the stress of high osmolarity, and HOGA MAPK in these mutants could not be phosphorylated under the stress of osmolality or oxidation (Furukawa et al., 2005). Similar to HOG1, Pbs2 was reported to wildly involve in stress responses in yeast and various pathogenic fungus (Gustin et al., 1998; Bahn et al., 2006; Cheetham et al., 2007). Bahn et al. (2005) reported that the human pathogenic fungus *Cryptococcus neoformans* Serotype A *hog1* and *pbs2* mutants are attenuated in virulence. The study from Cheetham et al. (2011) indicated that both *Candida albicans* with nonphosphorylatable Pbs2 and with mutation of the consensus sites of Pbs2 displayed the impaired stress resistance and attenuated virulence in a mouse model.

The development and mycotoxin bio-synthesis of fungi were also regulated by signaling pathways which interacting with MAP kinase cascade. By systematic disruption the MAP kinase genes in yeast, Kawasaki et al. (2008) found that kinase Ste20p and Ste50p interacted with G protein subunits. FadA is the alpha subunit of heterotrimeric G protein in *A. nidulans*, Hicks et al. (1997) reported that both asexual sporulation and ST (sterigmatocystin) production of *A. nidulans* require the inhibition of FadA-dependent signaling, which is also a conserved mechanism to regulate aflatoxins biosynthesis in aflatoxins producing fungi. Yu (2016) reported that the conidiation, vegetative growth, stress response, and toxigenesis of *A. nidulans* were governed by the components of G protein (including FadA, GanB, SfaD, and GpgA). And, (Shimizu and Keller, 2001) found sporulation was decreased in the *pkaA* (the cAMP-dependent protein kinase catalytic subunit, a downstream target of FadA) overexpression strain, as occurs in *fadA*-dominant active strains.

*A. flavus* distributes widely in the world as a pathogenic fungus. The pathogenicity, infection, and toxicity of *A. flavus* seriously impacts the safety of human society globally. It is very important to reveal the regulation mechanism of *A. flavus* pathogenicity. But the role of PbsB (Gene bank No. in NCBI: AFLA_083380) in the aflatoxin bio-synthesis and virulence of *A. flavus* keeps unknown. Therefore, this study was designed to explore the PbsB functions in mycelia growth, conidiation, sclerotia formation, aflatoxin production, and pathogenicity of *A. flavus*.

## Materials and methods

### Fungal strains and growth conditions

*A. flavus* strains used in the study were listed in Table 1. The primers used in the study were showed in Table 2. YPD (1% yeast extract, 2% peptone, 2% glucose, and 1.5% agar) and YES (2% yeast extract, 150 g/L sucrose, 1 g/L MgSO_4_·7H_2_O) were prepared for the cultivation of *A. flavus* strains in the study. Supplements (Uracil, and uridine) for auxotrophic marker (*pyrG*-) were added as required (Yang et al., 2016; Li et al., 2017). Strains were stored in 30% glycerol at −80°C.

**Table 1:** *A. flavus* strains used in this study.

**Strain name**	**Related genotype**	**Source**
CA14 Δ*ku70*Δ*pyrG*	Wild type (*pyrG-*)	Purchased from ATCC
CA14 Δ*ku70*	Wild type (*pyrG+*)	Prepared in our lab
Δ*pbsB*	Δ*ku70*, Δ*pbsB*::*pyrG*	This study
OE	Δ*ku70, pyrG*::*gpdA(p)*::*pbsB*	This study

**Table 2:** Primers used in this study.

**Primer name**	**Sequence (5′ → 3′)**
*pbsB*-AF	TAGTGCGTGCGTCCGTTTA
*pbsB*-AR	GGGTGAAGAGCATTGTTTGAGGCAGCGATGTCGCAAATCCAG
*pbsB*-BF	GCATCAGTGCCTCCTCTCAGACGCCTCCAAGGATGATGATGA
*pbsB*-BR	GAATGGTGTATCCGTAGTGC
*pbsB*-OrfF	ATCAGTGCAGCTCGAAGA
*pbsB*-OrfR	AGGCACCGTAGAAATCAA
*pyrG*-PF	GCCTCAAACAATGCTCTTCACCC
*pyrG*-PR	GTCTGAGAGGAGGCACTGATGC
*pyrG*-R	CAGGAGTTCTCGGGTTGTCG
*pyrG*-F	ATCGGCAATACCGTCCAGAAGC
OverlapF	CTGACATAGTCCATTGGCTG
OverlapR	GTTCCAGGTCATCTTCTTCG
*gpdA*-F	GCATCAGTGCCTCCTCTCAGACGTACAGTGACCGGTGACTCTTTCTGG
*gpdA*-R	GTGATGTCTGCTCAAGCGGGGTAG
OE-F	CTACCCCGCTTGAGCAGACATCACATGGCATCCGAAATCGATCCTGTAGC
OE-R	CCACCATCCATATACTCAACGCAGATGTAGAC
OE-overlap-F	AAGTCATAGAAGGATTCTGTTCGCCTACG
OE-overlap-R	TTATCCGTGACCCCATCGGAGCCA

### Bioinformatics analysis

The homologs of PbsB from (*A. flavus, A. oryzae, A. clavatus, A. fumigatus, A. niger, A. terreus, A. nidulans, N. crassa*, and *S. cerevisiae*) were downloaded from NCBI (http://www.ncbi.nlm.nih.gov), and further aligned with Clustal X. Protein domains of above 9 species were analyzed with InterPro (http://www.ebi.ac.uk/interpro/scan.html) and edited with IBS 1.0. Phylogenetic tree of PbsB homologs from these 9 species was constructed with MEGA5.1 by an algorithm of 1,000 times Neighbering comparison.

### Preparation of *pbsB* deletion and over expression strains of *A. flavus*

The *pbsB* deletion mutants were constructed according to Han et al. (2016) with minor modification. The deletion cassette was constructed by fusion PCR. In details, 5′- untranslated regions (5′-UTR) and 3′-UTR were amplified from *A. flavus* genomic DNA with two pairs of primers (*pbsB*-AF *and pbsB*-AR, *pbsB*-BF and *pbsB*-BR in Table 2). The intermediate *A. fumigatus pyrG* was amplified from *A. fumigatus* genomic DNA by primers *pyrG*-PF and *pyrG*-PR (Table 2) as transformation selecting marker. 5′-UTR and 3′-UTR of *pbsB* and *pyrG* were fused together by fusion PCR with nesting primers OverlapF and OverlapR (Table 2). Polyethylene glycol-mediated transformation of CA14Δ*ku70*Δ*pyrG* protoplasts was carried out as described by Cary et al. (2005). The *pyrG* prototroph strain (Δ*pbsB*: Δ*ku70*, Δ*pbsB*::*pyrG* showed in Table 1), in which the entire *pbsB* coding region was replaced by *pyrG*, was further tested with PCR (primers listed in Table 2), and the flanking regions of confirmed Δ*pbsB* strain was further sequenced by BioSune (Shanghai, China). The *pbsB* overexpression strains were prepared by homologous recombination (Nie et al., 2016). After four DNA fragments were amplified with four pairs of primers: *pbsB*-AF and *pbsB*-AR for 5′HR, *pyrG*-PF and *pyrG*-PR for *pyrG, gpdA*-F and *gpdA*-R for *gpdA(p)*, and OE-F and OE-R for 3′HR, they were overlapped with nesting primers: OE-overlap-F and OE-overlap-R (Table 2). And *pbsB* overexpression strains (OE) were prepared by Polyethylene glycol-mediated transformation of CA14Δ*ku70*Δ*pyrG* protoplasts with the overlap PCR production as mentioned above. The OE strains were further confirmed with primer *gpdA*-F and OE-R for 2,000 bp OE-ORF fragment, and with primer OE-overlap-F and OE-R for 4,730 bp OR-ORF fragment as shown in Table 2. Finally, the expression level of *pbsB* in *A. flavus* strains mentioned above was further assayed with qRT-PCR analysis.

### Quantitative reverse transcription polymerase chain reaction (qRT-PCR) analysis

The qRT-PCR was conducted according to the protocol provided by Zhang et al. (2016). Three micrograms of total RNA were treated with DNase I (Thermo Fisher Scientific, Waltham, MA, USA) to remove possible G-DNA contamination. One microgram G-DNA free RNA was reverse transcribed by using the Revert Aid First-strand cDNA Synthesis kit (Thermo Fisher Scientific, Waltham, MA, USA). qRT-PCR was carried out by using the SYBR Green Premix kit (Takara, Dalian, China) with Mx3000p thermocycler (Agilent Technologies). The expression levels of target gene were evaluated with the 2′ ΔΔCt method.

### TLC analysis of AFB1 production

The effect of PbsB in AFB1 production was performed after 5 d cultivation according to the method by Dhingra et al. (2013). 25 mL of liquid YES medium was inoculated with 10^4^ conidia/mL, and the cultures were shaking at 180 r/min at 28°C. The supernatant samples were analyzed by thin-layer chromatography (TLC). A volume of 3 μL supernatant was spotted onto a TLC plate (Si250F, J.T. Baker), and the plate was developed in acetone-chloroform (2:8, vol/vol), and dried at 80°C for 10 min, then the aflatoxin B1 was examined under UV light.

### Kernel assays

Peanut and maize kernels were prepared to discover the role of PbsB in plant infection following the method described by Yang et al. (2016) with minor modification. Each peanut and maize kernel was sterilized with 0.05% sodium hypochlorite. Viable cotyledon was dried and placed on sterile Petri dish plate. The cultures were incubated at 28°C in dark for 7 d. For AFB1 analysis, a Petri dish plate of kernels was soaked in double distill water for 10 h. After chloroform (half of the water volume) was added, the mixture was shacked at 180 r/min for 1 h, and kept still for another 2 h. The chloroform layer was collected and dried by evaporation. The sediment was re-suspended with 1 mL chloroform, and re-dissolved in 30 μL chloroform after dried by evaporation. Finally, 5 μL sample was analysis by TLC.

### Statistical analysis

The data in the study was presented as the means ± standard deviation (SD). The presence of statistical differences was determined by one-way ANOVA, and statistical significance was recognized when *P* < 0.05.

## Results

### Bioinformatics analysis of PbsB

**Figure 1:**
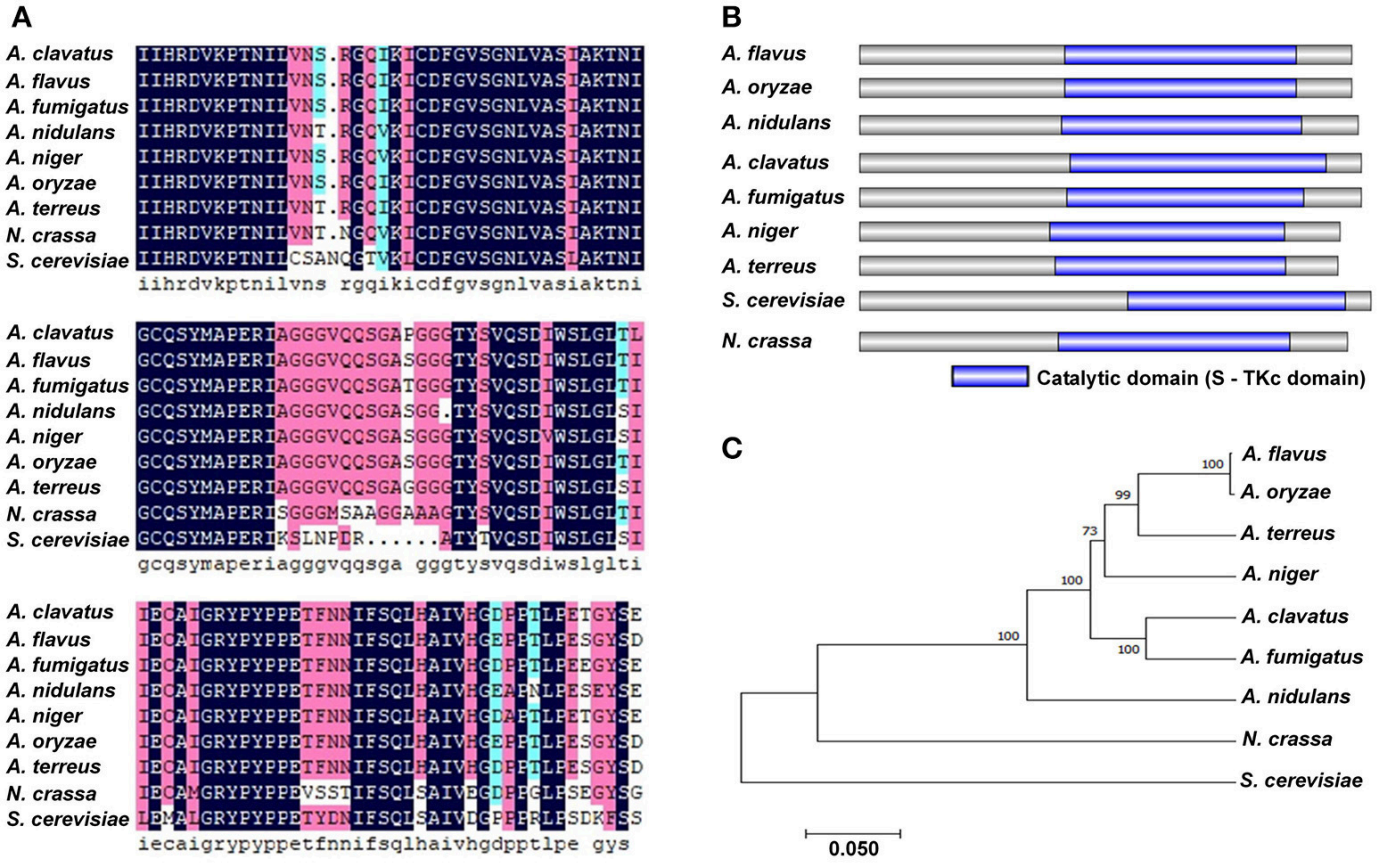
Bioinformatics analysis of PbsB. **(A)** Amino acid alignment of *A. flavus* PbsB and other 8 putative orthologs. Clustal X was used in this analysis. **(B)** Diagram shows the domains in PbsB among above 9 species. InterPro (http://www.ebi.ac.uk/interpro/scan.html) and IBS 1.0 were used in the analysis. **(C)** The diagram of the phylogenetic tree among 9 species.

PbsB protein in *A. flavus* and its orthologs in other 8 species were aligned by Clustal X, and the result showed that PbsB in *A. flavus* and *A. oryzae* had the highest similarity (83.27%), and the lowest homology was found between *A. flavus* and *S. cerevisiae* (29.70%) in Figure 1A. The protein domain in PbsB was further analyzed with IBS 1.0, and a catalytic domain (Serine/Threonine protein kinases) was found in all 9 species (Figure 1B), which meant that the catalytic domain was very conservative. Phylogenetic tree among these 9 species was further constructed with MEGA5.1 as shown in Figure 1C. The genetic relationship of PbsB from *A. flavus* and *A. oryzae* was found to be the closest, compared with the relationship of the protein from *A. flavus* and any other species, and the protein sequences of PbsB from *Aspergillus* spp. were classified into one cluster compared with the species from other genera (*N. crassa* and *S. cerevisiae*).

### PbsB in *A. flavus* enhances the growth of mycelia

**Figure 2:**
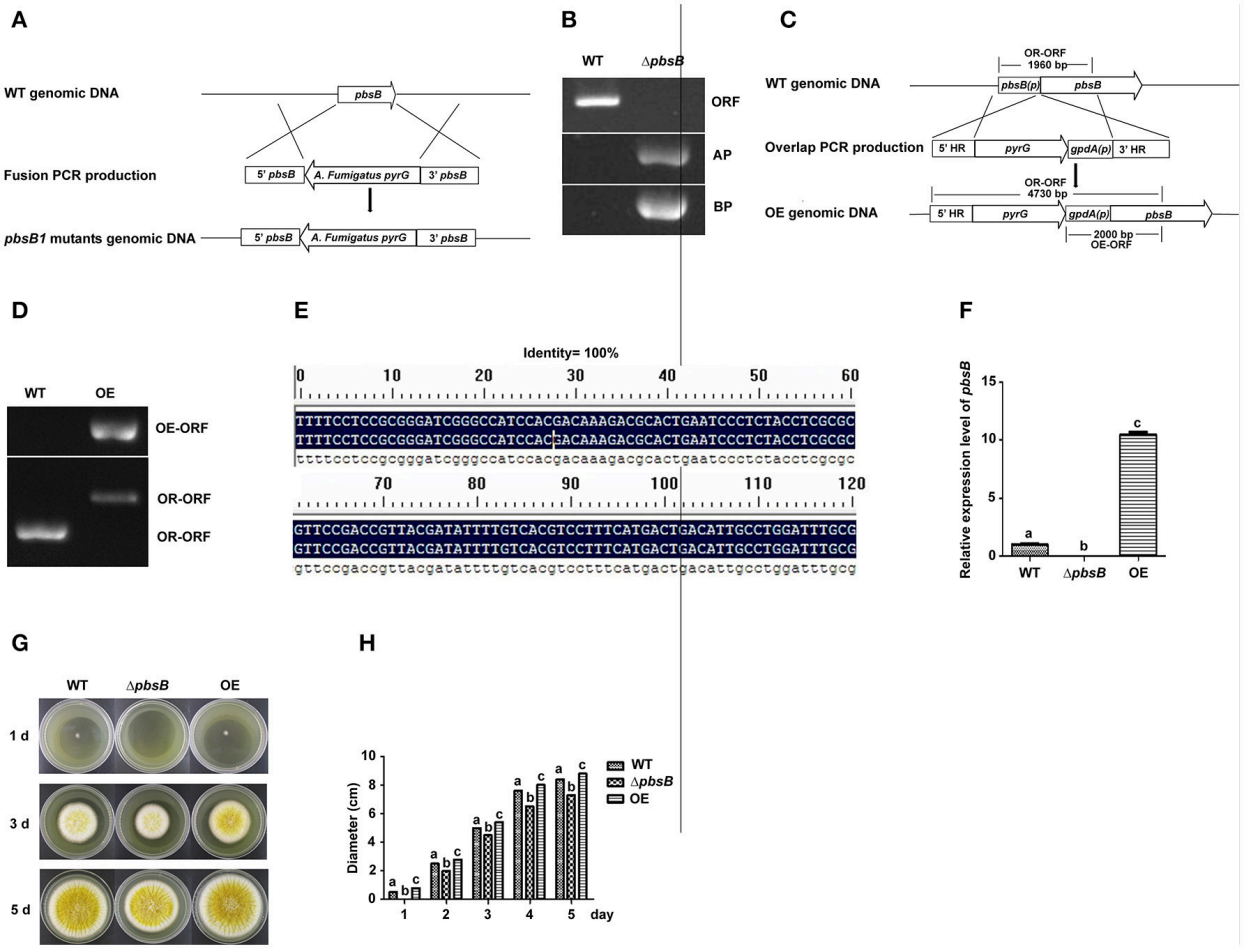
PbsB involved in the growth of *A. flavus*. **(A)** The scheme for Δ*pbsB* strain construction by homologous recombination. 5′-UTR (5′*pbsB*) and 3′-UTR (3′*pbsB*) of *pbsB* and 1.89 kb *pyrG* from *A. fumigatus* were amplified, respectively, and they were fused together with nesting primers. The *pbsB* deletion strain was prepared with *pyrG* to replace *pbsB* in WT (CA14Δ*ku70*Δ*pyrG*) through transformation the protoplast with the fusion production of 5′-UTR-*pyrG*-3′-UTR by homologous recombination. **(B)** The results of PCR analysis, in which ORF was amplified with primer *pbsB*-OrfF and *pbsB*-OrfR, AP was with *pbsB*-AF and *pyrG*-R, and BP was with *pyrG*-F and *pbsB*-AR. **(C)** The scheme for OE strain construction by homologous recombination. **(D)** OE strain was detected with PCR analysis. OE-ORF was amplified with primer *gpdA*-F and OE-R, and OR-ORF was with primer OE-overlap-F and OE-R. **(E)**. The alignment of flanking regions of *pyrG* and 5′ and 3′UTR of *pbsB* in Δ*pbsB* strain between sequencing results and constructed sequence map. **(F)** The expression level of *pbsB* in WT, Δ*pbsB*, and OE strain were analyzed with qRT-PCR. **(G)** Colony of WT *A. flavus*, Δ*pbsB* mutant and OE strains were cultured in YES medium for 1 d to 5 d. **(H)**. Comparative analysis of colony diameters, the letters “a,” “b,” and “c” used in the histogram of this study represented the significant difference among WT, Δ*pbsB* mutant, and OE strain (*P* < 0.05).

The *A. flavus pbsB* deletion strains (Δ*pbsB*) and over expression strains (OE) were constructed by transforming the protoplasts of WT (*pyrG-*) with the fusion PCR productions, respectively (Figures 2A,C), and the resulting transformants were confirmed by PCR analysis (Figures 2B,D). The result of PCR showed that the ORF of *pbsB* has been deleted from *pbsB* deletion mutants, and the amplification of AP and BP from Δ*pbsB* meant that ORF of *pbsB* has been replaced by *pyrG* from *A. fumigatus* (Figure 2B). The franking regions between *pyrG* and 5′-UTR and 3′-UTR of *pbsB* of the PCR confirmed Δ*pbsB* mutant were further sequenced and aligned with DNAMAN (Version: 6.0.40), the results (100% identity, Figure 2E) showed *pbsB* had been deleted as the scheme showed in Figure 2A. The results in Figure 2D showed that 2,000 bp DNA fragment (OE-ORF) only could be amplified from OE strain, and the OR-ORF fragment from OE strain was 4,730 bp, but it was only 1,960 bp for WT strain (Figures 2C,D). By qRT-PCR analysis, it was found that not *pbsB* activity could be detected in Δ*pbsB*, and the expression level of *pbsB* in OE was dramatically improved compared with WT (Figure 2F). These results showed that both Δ*pbsB* and OE strains of *A. flavus* were successfully constructed. In order to evaluate the role of PbsB played in the growth of mycelium, the conidia (10^4^ spores/mL) from *A. flavus* strains (WT, Δ*pbsB* and OE) were point inoculated onto YES agar in dark at 37°C for 5 d. The results showed that the colony diameter of Δ*pbsB* mutant was obvious smaller than that of WT from 1 to 5th d, and opposite situation was found in OE strain compared to WT strain (Figure 2G). The diameter of each mycelium colony was measured from 1 to 5 day, and the results of the histogram in Figure 2H revealed that the PbsB significantly improved the growth of *A. flavus*.

### PbsB up-regulates *A. flavus* conidation

**Figure 3:**
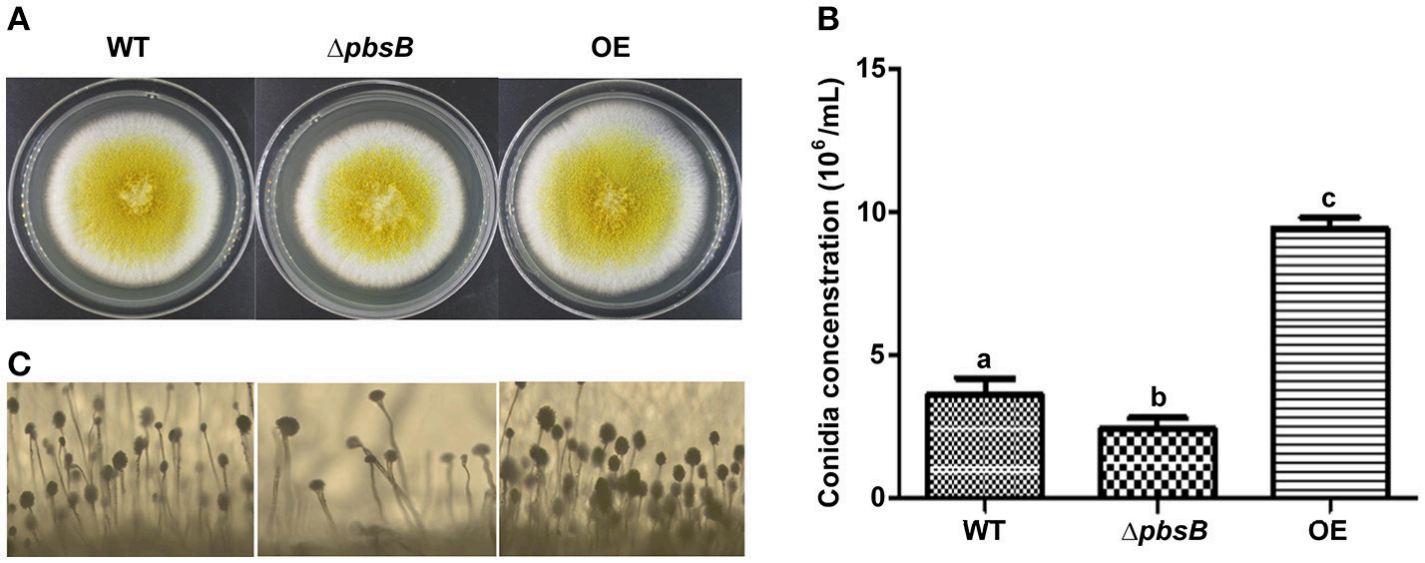
PbsB up-regulates *A. flavus* asexual reproduction. **(A)** Colony of WT, Δ*pbsB* mutant and OE strains were cultured in YES for 4 d. **(B)** The conidia numbers of WT and Δ*pbsB*, and OE strains were calculated with hemocytometer. **(C)** The conidiophores of WT, Δ*pbsB*, and OE strains were observed under microscope.

*Aspergillus* spp. always produces a massive number of conidia which are easily dispersed in the air in breeze. Conidiation is an important means to spread contamination for *A. flavus*, so the capacity of asexual reproduction—conidiation is a critical index to amplify the detriment of *A. flavus*. To explore the role of PbsB in conidiation, *A. flavus* strains (WT, Δ*pbsB*, and OE) were point inoculated onto YES at 37°C in dark, and phenotype was observed after 4 d cultivation. Δ*pbsB* mutant showed the decreased conidial production compared to WT strain (Figures 3A,B). Meanwhile, more conidiophores were observed in WT strain when compared to Δ*pbsB* mutant, and the conidiophores from Δ*pbsB* mutant were obvious dysplasia (Figure 3C). When *pbsB* overexpressed, significantly (*P* < 0.005) more conidia and conidiophores was observed in OE strain compared with WT strain (Figure 3). The result showed that PbsB improved the production of conidia, and the absence of PbsB significantly (*P* < 0.05) repressed the asexual reproduction of *A. flavus*.

### PbsB is important in the formation of sclerotia in *A. flavus*

**Figure 4:**
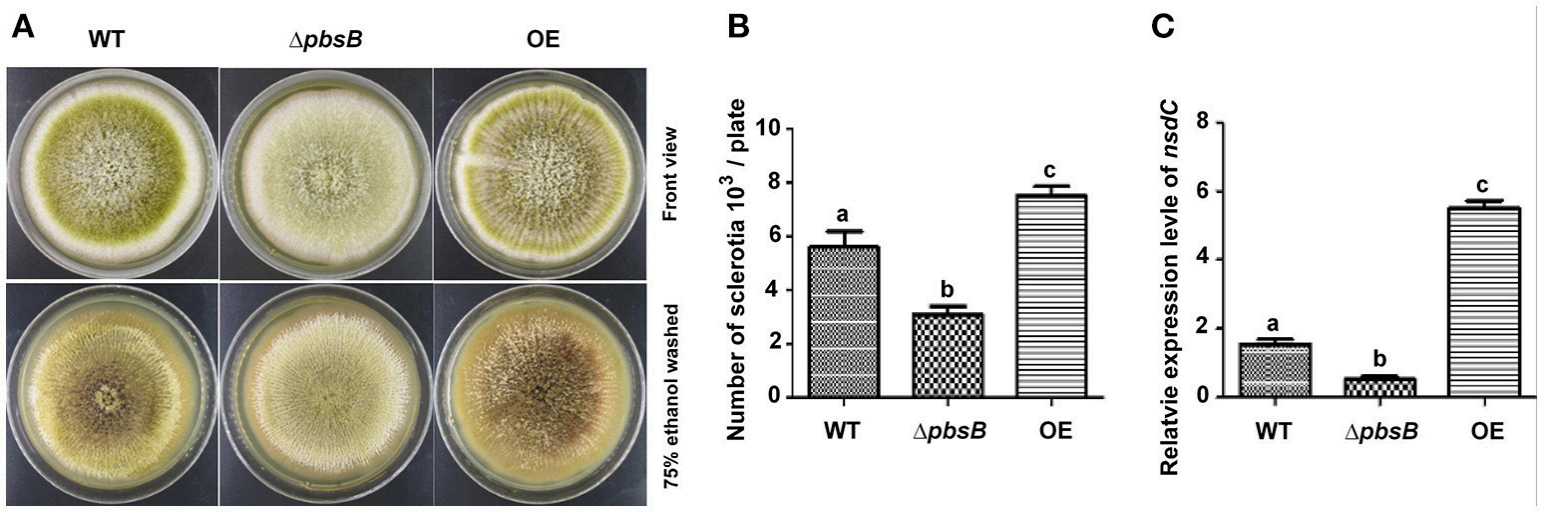
PbsB played an important role in sclerotia formation. **(A)** Point inoculated cultures of *A. flavus* WT, Δ*pbsB* mutant, and OE strain in YPD. Strains were cultivated for 7 d at 37°C. **(B)** The numbers of sclerotia in each plate were calculated. **(C)** The qRT-PCR analysis of gene expression levels of *nsdC* in WT strain, Δ*pbsB* mutant, and OE strain.

Sclerotia are readily produced by single strain of *A. flavus*, and sclerotia formation is commonly considered to be survival structure of *A. flavus* against unfavorable conditions. To reveal the bio-function of PbsB in the formation of sclerotia, *A. flavus* strains (WT, Δ*pbsB*, and OE) were point inoculated onto YPD at 37°C in dark for 7 d. After spraying with 75% ethanol, and the number of sclerotia on each plate was counted, respectively. The result showed that lack of PbsB significantly (*P* < 0.01) decreased the production of sclerotia, and the overexpression of *pbsB* obviously (*P* < 0.01) increased sclerotia production compared with WT (Figures 4A,B). The expression level of sclerotia regulator (*nsdC*) was also analyzed by qRT-PCR, and the results showed that *nsdC* was significantly (*P* < 0.01) down-regulated at 72 h when PbsB was absent, and was dramatically (*P* < 0.005) up-regulated when PbsB was overexpressed (Figure 4C). All these results indicate that PbsB is important in sclerotia formation in *A. flavus*.

### PbsB plays a vital role in AFB1 bio-synthesis of *A. flavus*

**Figure 5:**
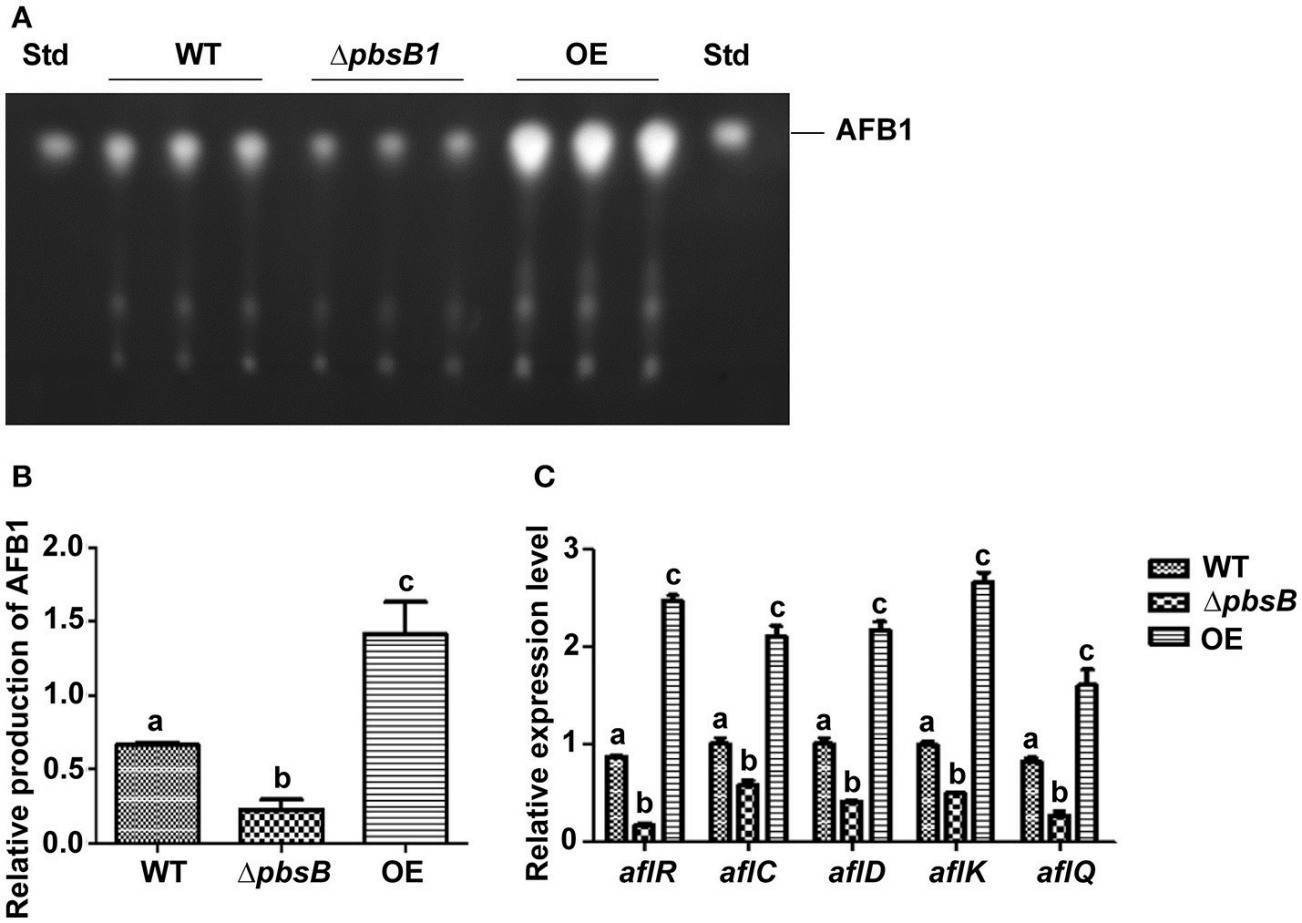
The role of PbsB in the AFB1 bio-synthesis of *A. flavus*. **(A)** TLC analysis of AFB1 production from WT, Δ*pbsB* mutant, and OE strain after 5 d of incubation. **(B)** Relative quantification of AFB1 production of *flavus* strain as mentioned in **(A)**. **(C)** Relative expression levels of aflatoxin bio-synthesis regulatory and structural genes *aflR, aflC, aflD, aflK*, and *aflQ* monitored by qRT-PCR at 48 h.

The role of PbsB in AFB1 production was analyzed by cultivating the WT, Δ*pbsB* and OE strains in the YES liquid medium, and the samples were collected at 5th day. The result of TLC analysis showed that AFB1 production was significantly (*P* < 0.005) decreased when *pbsB* was deleted, and the mycotoxin was obviously (*P* < 0.005) elevated when *pbsB* overexpressed in OE strain compared with WT strain (Figures 5A,B). The expression levels of aflatoxin bio-synthesis gene *aflC, aflD, aflK*, and *aflQ*, and regulatory gene *aflR* at 48 h were further analyzed by qRT-PCR, and the results showed that the expression levels of the aflatoxin bio-synthesis and regulatory genes were all significantly down-regulated in Δ*pbsB* mutant and up-regulated in OE strain compared to WT strain (Figure 5C). These results reflected that PbsB positively regulated the aflatoxin bio-synthesis through aflatoxin bio-synthesis and regulatory genes (*aflR, aflC, aflD, aflK*, and *aflQ*).

### PbsB involves in the resistance of *A. flavus* to hyperosmotic stress

**Figure 6:**
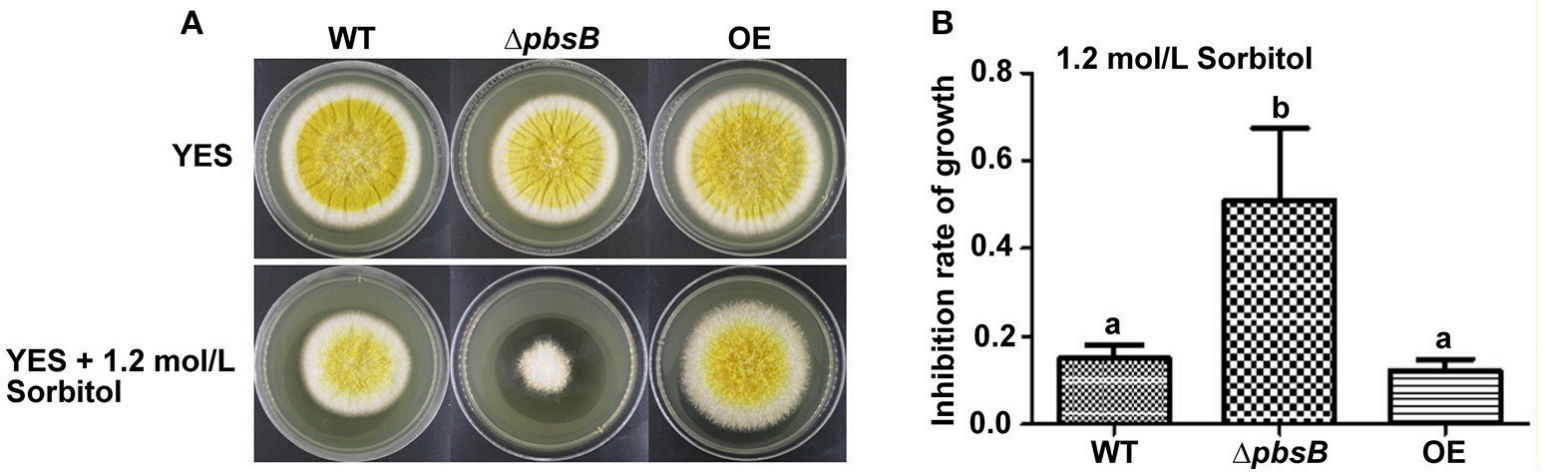
The Δ*pbsB* mutants were more sensitive to hyperosmotic stress**. (A)** The colonies of WT, Δ*pbsB* mutant and OE strain in YES medium with or without 1.2 mol/L Sorbitol. **(B)** Inhibition rate of growth with 1.2 mol/L Sorbitol. The inhibition rate = (diameter of colony without inhibitor—diameter of colony with inhibitor)/diameter of colony without inhibitor.

PbsB was reported to play an important role in the resistance of *C. albicans* to hyperosmotic conditions (Arana et al., 2005). To assess the role of PbsB under hyperosmotic stress in *A. flavus*, WT, Δ*pbsB* and OE strains were inoculated onto YES agar with 1.2 mol/L sorbitol for 4 d. It was found that the inhibition rate of Δ*pbsB* mutants dramatically (*P* < 0.01) greater than that of WT and OE strains (Figure 6). The results revealed that *pbsB* deletion mutant was more susceptible to hyperosmotic stress, and PbsB was one of the critical factors in *A. flavus* to fight against hyperosmotic conditions.

### PbsB is essential for *A. flavus* pathogenicity

**Figure 7:**
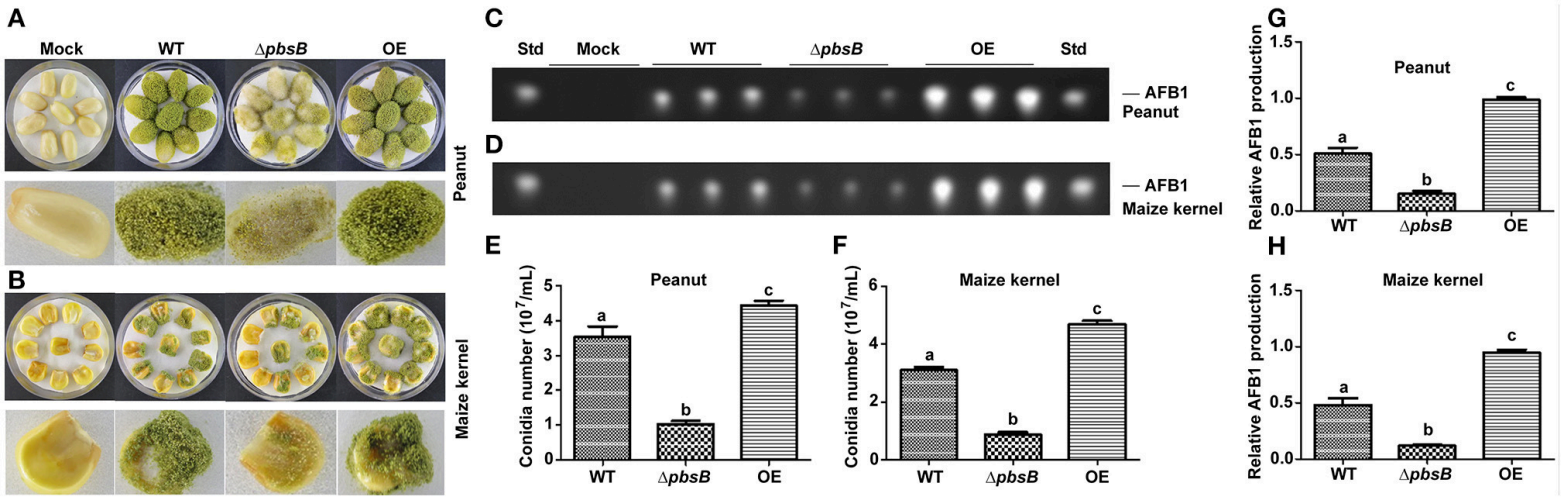
Crop grains colonization of WT, Δ*pbsB* mutant, and OE strain. **(A,B)** *A. flavus* strains cultured on peanut and maize kernels. **(C,D)** TLC analysis of AFB1 levels in infected peanut and maize kernels. **(E)** The number of conidia on peanut seeds counted after 7 d inoculation. **(F)** The number of conidia on maize kernel. **(G)** AFB1 level produced from *A. flavus* invaded peanut seeds from the results of **(C,H)**. AFB1 level produced from *A. flavus* invaded maize kernel from the results of **(D)**.

Peanut and maize kernels were inoculated with 10^4^ spores/mL of WT, Δ*pbsB* and OE strains in Petri dish plate for 7 d, respectively. It was shown in Figures 7A,B,E,F that dramatically (*P* < 0.005) reduced conidiation was observed in Δ*pbsB* mutant, and significant (*P* < 0.05) increased sporulation yield was found in OE strain compared to that in WT strain. TLC analysis showed that AFB1 bio-synthesis capacity of *A. flavus* on peanuts was significantly (*P* < 0.005) repressed when PbsB was absent from Δ*pbsB* mutant, and was obviously (*P* < 0.005) improved when PbsB was over-produced in OE strain (Figures 7C,G). To maize kernel, similar results were observed (Figures 7D,H). All these results of the study indicated that PbsB played an essential role in crops infection.

## Discussion

MAP kinase cascades are important pathway for yeast and filamentous fungus to respond to the stimuli from outside environment (Gustin et al., 1998; Furukawa et al., 2005; Bahn et al., 2006).

As an MAPKK, PbsB catalyzes the phosphorylation of MAPK under the stress of osmolality or oxidation (Furukawa et al., 2005). In *A. flavus, pbsB* (AFLA_083380) was made up by 2002 nucleotide residues, composing of 2 extrons and 1 introns, which were translated into protein PbsB (XP_002373253) with 643 amino acid residues. The bio-information analysis on PbsB showed that it harbored a conservative Serine/Threonine protein kinases domain, and the PbsB protein sequences in all selected *Aspergillus* spp. were grouped into one cluster, which suggested that the sequence of PbsB was very conservative, and it play a very important role in MAP kinase cascades.

PbsB involves in the growth of mycelium, conidiation, and sclerotia formation. Arana et al. reported 2005 that Pbs2 repressed hyphal formation in *C. albicans*, and deletion of *pbs2* from *C. albicans* increased hyphal growth under different conditions. On the contrary, our study found that PbsB in *A. flavus* positively participated the regulation of mycelium growth (Figures 2G,H). Liu et al. (2017) reported that vegetative growth of *Beauveria bassiana* Δ*pbs2* strains on minimal media with different carbon/nitrogen sources was suppressed, and suffered severe conidiation defects. In the study, sever asexual reproduction defects were also observed in *A. flavus pbsB* mutant (Figure 3,*P* < 0.05) on YES medium and on the surface of peanut and maize kernels (Figures 7A,**B**,**E**,**F**,*P* < 0.005). And it was also found that the overexpression of *pbsB* in OE strain significantly improved its conidiation capacity on both conditions (Figures 3,7). Cary et al. (2012) and Kim et al. (2009) reported that *nsdC* gene (encoding a putative transcription factor) was found up-regulating vegetative growth in *A. nidulans*. Our Q-PCR results for *nsdC* gene (Figure 4C) showed that when *pbsB* was deleted, the expression level of *nsdC* was down-regulated significantly (*P* < 0.01), and when *pbsB* was overexpressed, the activity of *nsdC* was up-regulated dramatically (*P* < 0.005), which meant that *pbsB* regulated the asexual reproduction (conidiation) of *A. flavus* at the upstream of *nsdC*. No reports on the role of PbsB in sclerotia development was found, yet. The formation of sclerotia is considered to survive the unfavorable conditions for *A. flavus* (Wicklow, 1987). In the study, we found that the absence of PbsB in *A. flavus* obviously (*P* < 0.01) down-regulated the formation of sclerotia, and the increase of PbsB in OE strain significantly (*P* < 0.01) improved sclerotia formation. It is reported that *nsdC* gene is also required for production of sclerotia (Cary et al., 2012). From the result of Figure 4, it was concluded that PbsB positively regulated the formation of sclerotia in *A. flavus* through *nsdC*. The results of our study disclosed that PbsB plays an important role in the mycelium growth, development, and reproduction of *A. flavus*.

PbsB up-regulates the bio-synthesis of AFB1 in *A. flavus*. No reports which directly linked the role of PbsB with production of mycotoxin is found till now. Our study on *A. flavus* revealed that the absence of PbsB dramatically &(*P* < 0.005) reduced the level of AFB1 production in liquid YES medium and on the surface of both peanut and maize kernels. And when the expression level of *pbsB* was up-regulated in OE, the production of AFB1 increased dramatically (Figures 5,**7**,*P* < 0.005). The expression of regulatory gene *aflR* is required for transcription of most structural genes in the aflatoxin gene cluster, and structural gene *aflC, aflD, aflK*, and *aflQ* are involved in the conversion of acetate to AFB1 in AFB1 bio-synthesis pathway (Yu, 2012). The results in Figure 5 showed that the expression level of *aflR, aflC, aflD, aflK*, and *aflQ* were significantly down-regulated in *pbsB* deletion mutant and up-regulated in OE strain, which reflected that PbsB increased AFB1 bio-synthesis level through up-regulating the expression levels of regulatory and structural genes in the aflatoxin gene cluster (Figure 5C). Our research revealed that PbsB took part in the regulation of AFB1 bio-synthesis at the up-stream of the orthodox pathway of aflatoxin bio-synthesis (such as *aflR* and *aflQ*). Further exploration of the bio-function of PbsB in mycotoxin synthesis in pathogenic fungus would reveal the role of MAP kinase cascades in secondary metabolism in filamentous fungus.

PbsB is an essential toxic factor to encounter severe environmental stresses in *A. flavus*. In the crop models of the study, attenuated toxicity of *A. flavus* was found when PbsB was absent. Δ*pbsB* mutant was found to produce significantly fewer conidia and AFB1, but more conidia and AFB1 were produced in OE strain when *pbsB* was overexpressed. When pathogenic fungi invade host plant, they face a serials of challenges from outside environment, including hyperosmotic conditions and peroxide stress. Arana et al. (2005) revealed that the human pathogen *C. albicans* lacking Pbs2 was defective in growth under hyperosmotic conditions mediated by sorbitol. Mkc1 is involved in the response to oxidative stress and cell wall integrity (Correia et al., 2017). And the Western blot signal of phosphorylated Mkc1 in response to hydrogen peroxide is reduced significantly in *pbs2* mutants (Arana et al., 2005). Esquivel-Naranjo et al. (2016)reported that *Trichoderma atroviride pbs2* mutants were highly sensitive to cellular insults, such as osmotic and oxidative stress, cell wall damage, high temperature, cadmium, and UV irradiation. Our results from the study showed PbsB played an important role in the resistance of *A. flavus* to severe outside environmental conditions, as well as to the environment inside its hosts.

The results of our study revealed the bio-function of *pbsB* in mycelia growth, conidiation, sclerotia formation, aflatoxin production, and pathogenicity of filamentous pathogenic fungus *A. flavus*, and might provide potential targets in the control of the contamination from pathogenic fungus.

## Author contributions

ZZ designs and writes the manuscript, takes part in all experiments of the whole project, and provided funds support. JY takes part in all experiments designs, and constructed *Aspergillus flavus* deletion strains in the study, took part in the experiments on stress resistance of *A. flavus*, and provided funds support. ZC took part in the experiments on aflatoxin production analysis, and morphogenesis analysis. FZ took part in the experiments of peanut seeds and corn grains model construction. JS took part in the experiments of corn grains model construction. SW took part in the designation of the project, manuscript correction, and provided funds support. ZG repeated all the experiments (from Figures 2–7), and analysis of the data for the work. DL prepared the over-expression strain and was responsible for the DNA sequencing of the flanking regions of the deletion strain. YZ took part in the preparation of the over-expression strain.

### Conflict of interest statement

The authors declare that the research was conducted in the absence of any commercial or financial relationships that could be construed as a potential conflict of interest.
